# Interactions of mitochondrial and skeletal muscle biology in mitochondrial myopathy

**DOI:** 10.1042/BCJ20220233

**Published:** 2023-11-15

**Authors:** Valeria Di Leo, Tiago M. Bernardino Gomes, Amy E. Vincent

**Affiliations:** 1 Wellcome Centre for Mitochondrial Research, Translational and Clinical Research Institute, Faculty of Medical Sciences, Newcastle University, Newcastle NE2 4HH, U.K.; 2 NIHR Newcastle Biomedical Research Centre, Biomedical Research Building, Campus for Ageing and Vitality, Newcastle upon Tyne NE4 5PL, U.K.; 3 NHS Highly Specialised Service for Rare Mitochondrial Disorders, Newcastle upon Tyne Hospitals NHS Foundation Trust, Newcastle upon Tyne NE2 4HH, U.K.; 4 John Walton Muscular Dystrophy Research Centre, Translational and Clinical Research Institute, Faculty of Medical Sciences, Newcastle University, Newcastle NE2 4HH, U.K.

**Keywords:** function, metabolism, mitochondria, mitochondrial dysfunction, structure

## Abstract

Mitochondrial dysfunction in skeletal muscle fibres occurs with both healthy aging and a range of neuromuscular diseases. The impact of mitochondrial dysfunction in skeletal muscle and the way muscle fibres adapt to this dysfunction is important to understand disease mechanisms and to develop therapeutic interventions. Furthermore, interactions between mitochondrial dysfunction and skeletal muscle biology, in mitochondrial myopathy, likely have important implications for normal muscle function and physiology. In this review, we will try to give an overview of what is known to date about these interactions including metabolic remodelling, mitochondrial morphology, mitochondrial turnover, cellular processes and muscle cell structure and function. Each of these topics is at a different stage of understanding, with some being well researched and understood, and others in their infancy. Furthermore, some of what we know comes from disease models. Whilst some findings are confirmed in humans, where this is not yet the case, we must be cautious in interpreting findings in the context of human muscle and disease. Here, our goal is to discuss what is known, highlight what is unknown and give a perspective on the future direction of research in this area.

## Our muscles and mitochondria

Volume wise, skeletal muscle tissue is mostly composed of long, cylindrical, multi-nucleated, and contractile cells, known as muscle fibres, which are responsible for the volitional function and high energy demands of skeletal muscles [1]. Skeletal muscle contains distinct types of muscle fibres, which are classified based on the most abundant myosin isotype (Myh7, Myh2 or Myh1). Fibre types are metabolically different; type I (or oxidative slow twitch) fibres express Myh7, have 2–3 fold higher mitochondrial content and lower capacity for ATP production via non-oxidative pathways, when compared with type II (or glycolytic fast twitch) fibres, which express either Myh2 or Myh1, or both [2]. However, a recent study identified ribosomal specialisation as the major driver of skeletal muscle fibre type diversity both in healthy and diseased skeletal muscle [3]. Many other characteristics define the type and function of muscle fibres, such as capillary density and blood flow through the tissue, efficiency of oxygen extraction from blood and oxygen fixation by myoglobin, myosin ATPase capacity, and twitch contraction time [4–7]. Healthy skeletal muscle consists of a mix of fibre types with the varying proportions of fibre types in a muscle closely correlated to its function and capacity [7].

As with other organs and cellular processes, skeletal muscle mass and strength are typically reported to decline as we age [8]. Remarkably, a reduction in mitochondrial mass [9] and increased mitochondrial dysfunction have been reported to only affect a small percentage of fibres within the aging muscle [10]. Whilst it is at present unclear whether changes in mitochondrial biogenesis, mitophagy, or both lead to the decline in mitochondrial mass, we know that the age-related dysfunction in mitochondrial oxidative phosphorylation (OxPhos) is caused by sporadic mitochondrial DNA (mtDNA) mutations, typically mtDNA deletions, which clonally expand in individual fibres over time [10]. A similar phenomenon either plays an important role or underlies the pathophysiology of some muscle diseases in humans, including some types of genetically determined mtDNA maintenance disorders, which present as mitochondrial myopathy, some neuromuscular disorders, such as inclusion body myositis [11,12], and iatrogenic muscle diseases caused by mitotoxic therapeutics, such as antiretrovirals [13,14].

**Figure 1.**
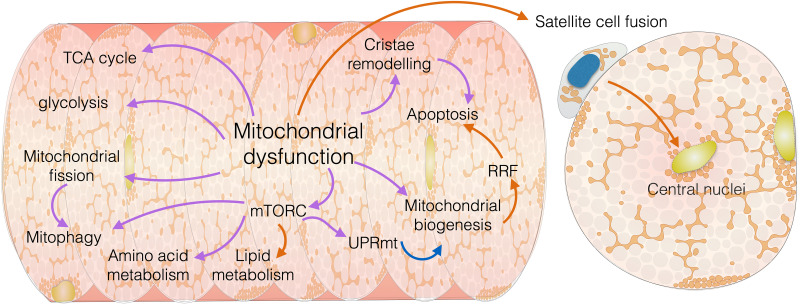
Schematic demonstrating the interconnected relationship of mitochondrial function with mitochondrial and muscle biology. Arrows are colour coded to indicate whether data supporting the relationship is from humans (Orange), animal models (Blue) or both (Purple).

In these conditions, mitochondrial dysfunction is only observed in a proportion of fibres while sparing others, and when examined longitudinally using histological or immunofluorescent techniques, mitochondrial dysfunction is found to only be present in small longitudinal segments, bordered by unaffected regions [10,15,16]. The accumulation and spread of mtDNA mutations and OxPhos dysfunction in skeletal muscle are covered elsewhere [17]. Here, we will focus on the interactions between mitochondrial dysfunction and muscle biology in mitochondrial myopathy (Figure 1). As our knowledge on such mechanisms in human tissue remains limited, we often must rely on model organisms where similar processes have been better characterised to gain insights. Whilst beneficial, we must be cautious when interpreting and translating such findings into research on human diseases, due to clear and significant differences between species, disease models and naturally occurring genotypes (Table 1).

**Table 1:** Highlight of most relevant data discussed in the review for the understanding of interactions between mitochondrial and skeletal muscle biology in the contest of mitochondrial myopathy

	Highlighted data	Organism	Reference
	*1. Metabolic remodelling*		
	Mitochondrial specialisation between fibre types	Human	[29]
	Proteomics changes between COX-negative and -positive fibres	Human	[30]
	CI subunits deficiency with m3243.A > G mutation	Human	[32]
	CI and CIV subunits deficiency with single, large-scale mtDNA deletion	Human	[21,33]
	CII and CV subunit increase with single, large-scale mtDNA deletion	Human	[33]
	Starvation-like response with multiple mtDNA deletions	Deletor mice, human,	[35,36]
	Mitochondrial integrated stress response with multiple mtDNA deletions	Deletor mice	[39]
	Remodelling of one-carbon pathways with multiple mtDNA deletions	Deletor mice, human	[40]
	Starvation-like response with m.8344A > G mutation and in COX10 deficiency	COX10 KO mice, human	[48,49]
	Metabolic remodelling in reversible infantile respiratory chain deficiency	Human	[53,54]
	Compensatory metabolism of lactate with severe mitochondrial myopathy phenotye	Ndufs4 KO mice, muscle specific type II fibres Mfn1/Mfn2 KO, human	[63,64,66,67]
	Succinate as a skeletal muscle remodelling modifier	Wild-type mice	[71,72]
	Rapamacin as an effective treatment	Muscle specifc Cox15 KO mice, Deletor mice, human	[39,135,141]
	Nicotinamide riboside and niacin as an effective treatment	Deletor mice, human	[41,47]
	Hypoxia as an effective therapy	Cells, zebrafish model, Leigh syndrome mice	[66,67]
	*2. Mitochondrial morphology*		
	Link between cristae morphology and cell specific metabolism	Worm, flies, mice, human	[83,84]
	Differential morphology and metabolism between subsarcolemmal and perinuclear mitochondria and intermyofibrillar mitochondria	Human	[86–88]
	Continuous mitochondrial network for efficient energy distribution	Wild-type mice	[91,92]
	Decreased cristae density with mitochondrial dysfunction	Cells	[99]
	Donut mitochondria, concentric cristae, paracrystalline inclusions with mitochondria dysfunction	Human	[97]
	Increased number of nanotunnels with mitochondria dysfunction	Human	[96,97]
	Mitochondrial network fragments with higher mtDNA mutation load	Cells, human	[84,93]
	*3. Mitochondrial turnover*		
	Fibre type switching and exercise intolerance	PGC-1α KO mice	[117]
	Decreased levels of oxidative phosphorylation and fatty acids oxidation	Surf1 KO mice, Sco2 KO/KIN, muscle-specific Cox15 KO, Deletor mice	[41,118,120]
	Activation of mitochondrial biogenesis after treatment	Surf1 KO mice, Sco2 KO/KIN, muscle-specific Cox15 KO, human	[41,47,118,122,123,124,125]
	Activation of perinuclear mitochondrial biogenesis in mitochondrial dysfunction	Human	[16]
	Activation of UPR^mt^ within the fibre and in the perinuclear area	Human	[16,41]
	AICAR as an effective treatment	KO/KI, muscle-specific Cox15 KO	[118]
	Bezafibrate as a potential effective treatment	Deletor mice, human	[122,123,124,125]
	Mitophagy and autophagy impairment with both mtDNA deletions and point mutations	Muscle-specific Cox15 KO, human	[130,133,134]
	Mitophagy impairment with stage-wise dynamics	Parkin KO flies, PINK1 KO flies, Deletor mice, human	[16,135,138]
	Restore of mitophagy and autophagy by rapamycin treatment	Muscle-specific Cox15 KO, Deletor mice, human	[39,134,135]
	Modulation of mTORC1 and mitophagy and rapamycin dose-dependency	Coq9^R239X^ mice	[140]
*4. Cellular processes*			
*Apoptosis*	Myofibres apoptosis in atrophy or myopathy	Mice models, human	[148]
	Correlation of apoptosis to high mtDNA mutation load and respiratory chain deficiency	Human	[149,150]
	High rate of apoptosis in myofibres displaying mitochondrial myopathy	Human	[149,151,152,156–158]
*ROS production*	Associated ROS over-production to oxidative phosphorylation defects	Ant1 KO mice, human	[153,166,169]
	Increased antioxidant enzymes to ROS over-production	Ant1 KO mice, human	[166–168]
	ROS over-production as a disease modifier for satellite cells, mitophagy, autophagy	Cells, mice, human	[172–175]
*Ca^2+^ signalling*	Impairment of Ca^2+^ uptake capacity due to decrease in porin	Human	[21,31–33]
	Modulation of TCA cycle by Ca^2+^ handling	Cardyomyocites, human	[177,178,184–187]
	CIV or CV deficiency linked to impaired mitochondria-ER contact sites, UPR^mt^ and UPR and different contraction patterns	Cells, Drp1 KO mice, human	[191–193,197,198]
*Novel mitochondrial process*	Excess of mtDNA copy number to sense and modulate mitochondrial homeostasis	Cells, Tfam^+^/^−^ mice	[199,200]
	mtDNA molecules extrusion under oxidative stress conditions, apoptosis or in mitochondrial myopathy	Cells, human	[97,202,203]
	mtDNA detection in a cell-free state with paracrine/endocrine role	Human serum and plasma	[208]
	Mitochondrial-derived vescicles for mitochondrial turnover and regenerative potential in skeletal muscle	Cells, mice models, human	[209–212]
	*5. Muscle cell morphology and function*		
	Impact of mitochondrial morphology onto myofibrillar morphology and branching	Wild-type flies	[214]
	Link bewteen size/position of mitochondria and the cross-sectional area of myosin fibrilis/muscle	Wild-type flies, wild-type mice and rats, human	[216,218]
	Sarcolemmal distension and disruption of myofibrillar organisation in ragged-red fibres due to increased subsarcolemmal and intermyofibrillar mitochondria	Human	[97]
	Disruption of myofibrils and skeletal muscle dysfunction in mitochondrial dysfunction	Human	[217]
	Mitochondrial fusion and fission reduction with sarcopenia	Rats, muscle-specific Opa1 KO mice, human	[30,219,220]
	High level of mtDNA in mitochondria dysfunction only in a small portion of muscle fibres with atrophy	Deletor mice, rats, human	[222,223,224]
	Central nuclei and nucleophagy adjacent to clusters of lysosomes, mitochondria and mitolysosomes	Human	[135,225]

## Metabolic remodelling

It has long been known that under certain circumstances, cells can shift ATP production from mitochondrial OxPhos to glycolysis, with lactic acid fermentation in the cytosol, and decreased oxygen consumption, even in aerobiosis. Known as Warburg effect [18], this metabolic shift has been extensively studied in cancer cells and is associated with increased cancer aggressiveness and markers of mitochondrial dysfunction, including mtDNA mutations and depletion, and decreased mitochondrial mass and OxPhos capacity [19]. These changes are proposed as critical pathomechanisms essential to the progression of the cancer. A similar metabolic switch capable of rescuing energy production in tissues with OxPhos dysfunction is often hypothesised, but the evidence for such a mechanism in skeletal muscle affected by mitochondrial dysfunction remains lacking [20,21]. Although some studies suggest a positive correlation between OxPhos dysfunction and predominance of glycolytic type II fibres [22,23], other observations point towards a positive correlation with increased proportion of oxidative type I fibres [24–27]. However, these discrepancies may be due to differences in the age of participants, the metabolic profile determined by the type of skeletal muscle sampled, and the assay and analysis used to determine fibre type in these studies.

Aside from OxPhos, recent work suggests that other key metabolic pathways are altered in mitochondrial myopathy. The complexity of the muscle fibre proteome has been explored between the different fibre types using single-fibre proteomics, which revealed fibre type-specific profiles with important differences in structural and metabolic components, but also in mitochondrial proteins and pathways involved in adaptive responses [28,29]. A similar study compared single-fibre profiles between cytochrome *c* oxidase (COX)-positive and -negative skeletal muscle fibres of patients with mitochondrial myopathy [30]. Murgia et al. [30] demonstrated that COX-negative fibres harboured lower levels of OxPhos subunits from complexes I–V (CI-V). Lower levels of CI and CIV subunits have been similarly reported in single-fibre immunofluorescence and imaging mass cytometry studies [21,31–33]; however, in skeletal muscle from patients carrying single, large-scale mtDNA deletions, only proteins impacted by the mtDNA mutation display lower levels, whereas unaffected mtDNA-encoded proteins are overexpressed, possibly due to a compensatory effect [33]. Remarkably, glycolytic enzymes were only found to be higher in type II fibres, regardless of their COX status, suggesting that this is linked to fibre type-specific structural and metabolic differences, and it unlikely represents a rescue mechanism in energy deficient COX-negative fibres [29,30]. Nevertheless, it contrasts with observations from single-fibre proteomic studies suggesting that, during the aging process, proteins of glycolysis and glycogen metabolism are up-regulated in type I fibres and down-regulated type II fibres [34]. Interestingly, proteins involved in tricarboxylic acid (TCA) cycle and fatty acid oxidation were up-regulated in COX-negative fibres of both types, compared with COX-positive counterparts, supporting the role of these pathways in driving fibre type-independent metabolic compensatory mechanisms in mitochondrial myopathy [29,30].

Mitochondrial DNA mutations have been demonstrated to induce a starvation-like response in skeletal muscle of the Deletor mouse model, which carries a *TWNK* mutation [35], by up-regulating transcription of genes involved in amino acid (MTHFD2, SFPQ/PSF, ASNS, PSAT1) and lipid metabolism (FGF21) [36]. The Deletor mouse model mimics the skeletal muscle pathology and clinical phenotype of mtDNA maintenance disorders in humans, which present with slowly progressive muscle disease [37,38] with underlying transcriptional, translational and metabolic stress responses, usually referred to as integrated mitochondrial stress response (ISRmt) [39]. The mTORC1 complex is considered a central modulator of the ISRmt, as it orchestrates tissue remodelling by activating anabolic one-carbon pathways, such as the folate cycle, *de novo* serine biosynthesis, amino acid trans-sulfuration and dNTP synthesis [39,40]. Of importance, the mTORC1 signalling cascade network also modulates the amino acid starvation response [41] and the mitochondrial unfolded proteins response (UPRmt) [42]. In mitochondrial myopathies, these signalling pathways become chronically induced in skeletal muscle as the disease progresses, culminating with the release of the muscle-derived hormones, or mitokines, FGF21 and GDF15 [39,40,43–45], which are now established biomarkers for muscle presenting with mitochondrial OxPhos deficiency, mitochondrial translation and mtDNA maintenance disorders [45,46]. Furthermore, data from investigations on the Deletor mouse model strongly suggest that the skeletal muscle metabolome is altered compared with control animals, but could be rescued either by rapamycin treatment, a well-established mTORC1 inhibitor [39], or by niacin or nicotinamide riboside supplementation, which are water-soluble precursors of vitamin B_3_ found to down-regulate the mTOR signalling pathway [41,47].

Metabolic remodelling mechanisms in skeletal muscle have also been described in the muscle-specific *Cox10* knockout (KO) mouse model [48] and patients with mitochondrial myopathy due to the m.8344A > G mtDNA point mutation [49]. Patients show increased amino acid catabolism with up-regulated glutamate oxidation and alanine release into the circulation through remodelling of the TCA cycle [49]. The *Cox10* KO mouse model presents with these alterations suggesting that an increased glutamate flux through the TCA cycle follows disease progression and is an adaptive response to mitochondrial dysfunction in the muscle [49]. These changes are similar to the muscle physiological response to starvation under the control of mTORC1 [50,51] and evolve with disease progression, leading to the systemic release of FGF21, as an early event of the ISRmt, to induce systemic metabolic remodelling, including increased lipolysis in white adipose tissue, and hepatic gluconeogenesis, ureagenesis and ketogenesis [49]. However, some of these adaptations, such as increased lipolysis, proteolysis and gluconeogenesis become maladaptive in the *Cox10* KO mouse model and m.8344A > G patients due to OxPhos impairment and may contribute to their toxicity [49].

An adaptive metabolic remodelling, rewiring mitochondrial dysfunction and leading to complete or partial recovery of patients, has also been well documented to spontaneously occurs in the ultra-rare reversible infantile mitochondrial diseases [52]. The reversible infantile respiratory chain deficiency (RIRCD) is the most common and causes severe infantile metabolic myopathy, with patients requiring extensive life-sustaining measures, but often with full and spontaneous recovery, if they survive this metabolic crisis [53]. RIRCD is a remarkable digenic condition that requires patients to carry a m.14674T > C/G mt-tRNA^Glu^ mutation at homoplasmic levels, plus an additional heterozygous variant in a nuclear gene, either involved in glutamine/glutamate amino acid metabolism, such as *GOT2*, *GLS*, or in mt-tRNA^Glu^ and mt-tRNA^Gln^ metabolism, required for mitochondrial protein translation, such as *EARS2*, *TRMU* or *QRSL1* [54]. It has been shown that in RIRCD derived-myoblasts lines with TRMU deficiency, l-cysteine supplementation can rescue the OxPhos deficiency, highlighting a direct regulatory mechanism orchestrated by the amino acid metabolism [55]. Moreover, the events characterising the spontaneous recovery of respiratory chain deficiency in the skeletal muscle of RIRCD patients could be divided into three major phases over the course of the first year of the patient's life since birth [54]. Initially, metabolic rescue is driven by the activation of the ISRmt, which induces the secretion of both mitokines FGF21 and GDF15, and the activity of the transcriptional factors ATF5 and ATF4, which are responsive to the status of the amino acid metabolism in skeletal muscle [44]. Subsequently, mTOR activation, mediated by decreased levels of DEPTOR [56], induces the activation of mitochondrial biogenesis, which is supported by increased levels of PGC-1α and by increased protein synthesis and cell proliferation. Finally, the recovery phase is characterised by the restoration to baseline levels of mitochondrial stress markers with the expansion of healthy mitochondria in the skeletal muscle of RIRCD patients [54].

Mitochondrial metabolism produces metabolites, including acetyl-CoA and α-ketoglutarate, which are involved in epigenetic changes that modulate gene expression in the nucleus [57–59]. Imbalances in mitochondrial metabolism can cause impairment of signalling pathways and gene expression. In diagnostics, blood samples from patients with mitochondrial disease are routinely tested for lactate level and lactate/pyruvate ratio, where both metabolites are derived from glycolysis [60]. Some types of mitochondrial myopathy present a markedly differential lactate turnover in skeletal muscle due to OxPhos deficiency caused by either mtDNA or nuclear mutations [61,62]. In health muscle, OxPhos is the main source of ATP, but in the presence of high mtDNA mutation loads or OxPhos dysfunction, the muscle is more reliant on glycolysis, which leads to increased pyruvate production and reduction in NAD^+^ to NADH. Mitochondrial OxPhos is ultimately the main pathway driving pyruvate and NADH oxidation in healthy muscle; thus, in OxPhos-deficient muscle, NADH is oxidised to reduce pyruvate to lactate, which is released into the blood stream, while regenerating cytosolic NAD^+^ to sustain glycolytic ATP production and by-passing mitochondrial OxPhos [63]. Surprisingly, glycolysis was shown to be down-regulated in OxPhos-deficient muscle from myopathic patients, with either *TK2* mutations or RIRCD, as well as from mice with Mfn1 and Mfn2 conditional deletion in muscle type II fibres, as a consequence of loss of constitutive HIF1α signalling, down-regulation of the purine nucleotide cycle enzyme AMPD1, and activation of AMPK [64]. Remarkably, by combining *in vivo* isotope tracing with mitochondrial lactate uptake inhibition experiments, the authors demonstrated that lactate transport into mitochondria was essential to keep the steady state of TCA cycle intermediates, with inhibition of lactate transport into mitochondria compromising the lifespan of the mice [64]. However, this study focused on a very rare and often severe form of mitochondrial myopathy, and it remains unclear if this unexpected mode of metabolic reprogramming could play a role in mitochondrial myopathies in general.

Moreover, in patients, mitochondrial myopathy is characterised by a mismatched oxygen delivery and utilisation during exercise, which is explained by an increased capillary growth, induced by impaired muscle OxPhos, and leads to an increased blood flow to OxPhos-deficient fibres [65]. At present, it is not clear whether mitochondrial myopathy metabolic remodelling is activating skeletal muscle angiogenesis in a hypoxia-independent manner [65], or if hypoxia could induce metabolic remodelling in OxPhos deficient skeletal muscle. Recent studies have observed that hypoxia can slow the disease progression and prevent neurodegeneration in *Ndufs4* KO mice, having a direct impact on the activity of enzymes involved in the metabolism of both lactate and pyruvate, such as PDK1, PDH and LDHA [66,67]. Therefore, the extent of systemic accumulation of lactate in mitochondrial myopathies is driven by the proportion of OxPhos-deficient fibres and, can not only directly determine physical performance and response to exercise [68], by fuelling the TCA cycle for energy production [69], but also cross-talks with the epigenome via histone lactylation, a recently identified histone modification involved in metabolic regulation of gene expression [70].

Succinate is the substrate of succinate dehydrogenase, which is both involved in the TCA cycle and in CII of the mitochondrial respiratory chain, thus making it a key metabolic signal. Succinate has been shown to participate in the nutritional and exercise-induced fibre type remodelling from glycolytic type II to oxidative type I fibres through both Erk1/2 and SUCNR1 signalling pathways [71,72] with downstream modulation of calcium (Ca^2+^) and AMPK signals, regulating fibre type-specific gene expression profile [73,74]. Interestingly, some patients with mitochondrial myopathy with succinate dehydrogenase and aconitase deficiency have been reported to express changes in the synthesis and processing of iron-sulfur clusters proteins [75]. Furthermore, α-ketoglutarate, a TCA cycle intermediate, and S-adenosylmethionine, a product of the serine metabolism [43], together cover a prominent epigenetic role by regulating demethylation and methylation of nuclear DNA, respectively. Although epigenetic changes have yet to be fully characterised in patients with mitochondrial myopathies, it is expected that future studies will identify novel epigenetic mechanisms with a central role in the pathophysiology of these conditions, since there is strong evidence that both the TCA cycle and one-carbon metabolism can be impaired in mitochondrial myopathy [76].

Since the discovery and characterisation of mitochondrial diseases, evidence of metabolic remodelling in skeletal muscle has played an important role in the diagnosis of mitochondrial myopathy patients. For example, skeletal muscle biopsies can be obtained from patients and COX/SDH histochemistry can be used to assess respiratory chain dysfunction, while Gömöri trichrome staining can identify the presence of ragged-red fibres, a common pathological hallmark of mitochondrial myopathy [77,78]. Such histological markers of mitochondrial metabolic dysfunction have been a key part of the diagnostic pipeline [79], however, more recently, a genetics-first approach based on clinical presentation has reduced the need for such tests [80].

### Mitochondrial morphology

Mitochondrial function and morphology are intricately linked, and mitochondrial response to cell stressors are complex due to their structure and dynamics. Mitochondria have an outer mitochondrial membrane (OMM) and inner mitochondrial membrane (IMM) with the intermembrane space between them, and the matrix inside the IMM [81]. The IMM is highly folded to form cristae, which protrude into the matrix increasing the surface area of the IMM and therefore allowing for many more OxPhos complexes to be supported along the cristae membrane [82]. Cristae morphology is different across cell types and is supposed to be linked to mitochondrial metabolic properties [83,84]. Furthermore, mitochondria, once thought of as bean shaped organelles, are a dynamic network that undergoes fission and fusion that constantly change mitochondrial size and branching.

What happens to mitochondrial morphology upon mitochondrial stress or dysfunction? Data from cell cybrids carrying the m.3243A > G variant, show that as the mutation level increases from 50% to 90%, the mitochondrial network becomes more connected, whereas if the mutation load continues to increase towards 100%, OxPhos dysfunction increases and the mitochondria become more fragmented [85]. However, both structure and organisation of mitochondria are significantly different between skeletal muscle fibres and cultured cells. In skeletal muscle fibres, we can define three sub-classes of mitochondria: subsarcolemmal mitochondria residing just below the cell membrane [86], perinuclear mitochondria surrounding the myonuclei [87], and intermyofibrillar mitochondria sandwiched between the myofibrils [86]. The subsarcolemmal and perinuclear mitochondria are often more spherical, whereas the intermyofibrillar mitochondria can be quite branched [87,88]. Work in mouse skeletal muscle has shown that mitochondria are not transported as much around the cell but have regular fission and fusion events [89,90]. Furthermore, the connectivity of the mitochondrial network is a property that is dependent on fibre type, with greater fusion and mitochondrial network connectivity in more oxidative myofibres [90].

Other studies in mouse skeletal muscle using focused ion beam scanning electron microscopy have reported that the mitochondrial network is one continuous reticulum [91] and that this allows for energy distribution along the network [92]. It was further demonstrated that proactive and reactive network changes are designed to limit the spread of mitochondrial dysfunction [93]. It is therefore interesting to consider the potential benefits of increasing mitochondrial fusion at low levels of mitochondrial stress, both diluting the mtDNA mutation load [94]. If this is the case for fibres with lower mutation loads, it would suggest that the mechanisms proposed by Glancy et al. [93] may only become active when the burden of mitochondrial dysfunction becomes too high, which interestingly matches with findings in cybrid cells [85].

In human skeletal muscle, we would expect the same relationship between morphology and function that we observe in the cybrid cells. Certainly work that used 3D reconstructions to look at mitochondrial morphology in muscle of three related patients with the m.8344A > G variants, found that the individual with 40% mutation load had highly connected mitochondria when compared with healthy controls, whereas the individual with 60% mutation load had fragmented mitochondria as did the patient with 96% mutation load [87]. The challenge is that these mutation loads are from whole muscle homogenate containing a mix of cell types and different levels of mutation. As such, when morphology is examined at a single cell level, it is unrealistic to dissect what the mutation load and therefore mitochondrial function would be in this specific cell. To overcome these limitations, techniques that can look correlatively at mitochondrial function and morphology in single cells are needed [95].

It is further interesting to note that in muscle biopsies from patients with mitochondrial dysfunction, there is an increase in small mitochondria and structures known as mitochondrial ‘nanotunnels', such that these two measures can be used to distinguish between biopsies of controls and patients [87]. Mitochondrial nanotunnels are thin double membrane projections that connect adjacent or more distant mitochondria and are thought to be a stress response due to the restricted mitochondrial movement in skeletal muscle [96]. The increased number of small mitochondria in patients is likely due to increased mitochondrial fission because of mitochondrial dysfunction; however, mitochondria under stress conditions or dysfunctional are also known to swell and exhibit substantial ultrastructural changes [97]. Concentric cristae, paracrystalline inclusions, and other changes in cristae morphology and density, are all commonly associated with mitochondrial dysfunction. Furthermore, it is known that cristae density is increased in response to higher metabolic demand in exercised skeletal muscle [98], whereas it often becomes decreased with mitochondrial dysfunction [99]. Furthermore, cellular stress or mitochondrial dysfunction that is severe enough to trigger apoptosis, lead to changes of cristae ultrastructure that allow the release of cytochrome c to trigger a signalling cascade that activates apoptosis [99,100].

Donut mitochondria, which appear to have fused with themselves to form a ring, have been observed in muscle fibres of patients with mitochondrial dysfunction as well as in models of mitochondrial dysfunction [97]. *In silico* modelling has suggested that donut morphology could be a stable and easily reversible mitochondrial response to stress in alternative to apoptotic processes [101]. However, more recent and higher resolution work examining what appeared to be donut mitochondria, found that this may indeed be a sickle shape rather than having a hole all the way through. It is unclear what the benefit of the sickle shape may be and whether truly donut shape mitochondria exist, or if all donut mitochondria are in fact sickle-shaped mitochondria.

Finally, mitochondrial morphology itself appears linked to the metabolic and functional properties of mitochondria with more fused and branched mitochondria, such as those found in the intermyofibrillar having a higher capacity for ATP production compared with smaller, spherical mitochondria found in the subsarcolemmal [88]. We also know that mitochondrial fission is needed for the selective removal of mitochondria via mitophagy. As such, mitochondrial morphology contributes to the turnover and quality control of mitochondria to maintain a functional pool of mitochondria within the cell. The impact of reduced fission is illustrated by mutations in the *OPA1* gene, which have been associated with accumulation of mtDNA mutations, presumably due to impaired removal of mitochondria via mitophagy [102].

### Mitochondrial turnover

Mitochondrial turnover is dictated by the fine balance between biogenesis and degradation of mitochondria [103] and, because it is independent from the cell cycle, it takes place even in post-mitotic cells, including skeletal muscle fibres, with tissue-specific turnover rates [104]. Mitochondrial biogenesis is regulated by a group of transcriptional factors (e.g. PGC-1α, TFAM and NRF1), which control the expression of genes essential for mtDNA replication and transcription, import of nuclear encoded proteins, protein quality control, and augmentation of OxPhos function [105–107]. Indeed, healthy skeletal muscle fibres containing damaged mitochondria alongside other damaged organelles can be degraded by autophagy, while individual mitochondria can be selectively removed via mitophagy through different pathways [108]. We have known for decades that mtDNA mutations clonally expand in tissues, with random genetic drift being the accepted mechanism for the clonal expansion of mtDNA point mutations in mitotic cells [109,110]. However, many theories have also hypothesised that different selective mechanisms and pressures may be activated [16,111–114]. One such pressure is mito-nuclear signalling, which has been suggested to increase mitochondrial biogenesis [16]. Understanding mitochondrial turnover and the way it relates to mitochondrial function is crucial, since turnover rates may either facilitate the spread of mutated mtDNA molecules through biogenesis, or spare them from degradation, either way leading mutations to clonally expand over time within the tissue.

PGC-1α is usually described as the mitochondrial biogenesis master regulator and its activation can be achieved in different ways depending on the cellular energetic status [106,115,116]. When the AMP/ATP ratio increases, AMPK is activated and triggers a phosphorylation cascade that induces the activation of PGC-1α. Similarly, when the NAD^+^/NADH ratio increases, AMPK activates Sirt1, which deacytelases PGC-1α increasing its active forms. In both situations, the activated PGC-1α directs a boost of catabolic pathways, in particular inducing increased fatty acid oxidation and mitochondrial respiratory chain function. Moreover, a muscle-specific PGC-1α KO mouse model has been shown to express changes both in skeletal muscle pathology, including switching from oxidative to more glycolytic fibre type, and in skeletal muscle function, such as decreased exercise tolerance, which altogether are major features underlying metabolic adaptations in patients with inflammatory myopathies [117].

For these reasons, the hypothesis that targeting PGC-1α and/or activating mitochondrial biogenesis could overcome the effect of mtDNA or nuclear mutations by rescuing the phenotype of mitochondrial myopathy has been explored for decades but mainly in mice models. To test this, Viscomi and colleagues performed an elegant study where three different recombinant mouse models (Surf1 KO, Sco2 KO/Knock-in (IN), muscle-specific Cox15 KO) with COX deficiency in skeletal muscle, therefore presenting mitochondrial myopathy [118] were tested with 5-aminoimidazole-4-carboxamide ribonucleoside (AICAR), which is an AMPK agonist [119]. After 1 month of treatment, all three mice models presented with normal mtDNA content, normal creatine kinase activity in skeletal muscle, increased level of both CIV subunits (COX1 and COX5a) and fatty acids oxidation related genes (CD36/FAT) [118]. Similar observations were gathered when treating the Deletor mouse model with the pan-PPAR agonist, bezafibrate, which has long been thought to activate PGC1-α [120]. Indeed, treated mice showed a decreased accumulation of both multiple mtDNA deletions and COX-deficient fibres in skeletal muscle, and a decrease in FGF21 expression [120]. Surprisingly, mitochondrial biogenesis was not activated in the Deletor mouse after bezafibrate treatment, although initial observations in patients with mitochondrial myopathy reported amelioration in fatty acid oxidation [121,122] and respiratory chain function [123]. In a recent open-label observational experimental study of six patients with the m.3243A > G mutation, mitochondrial biogenesis was minimally induced by bezafibrate treatment even at doses higher that those used to treat dyslipidaemia; rather an increase in serum mitochondrial mitokines FGF21 and GDF15 was observed together with dysregulation of both fatty acid and amino acid metabolisms [124].

Indeed, the activation of mitochondrial biogenesis and the rescue of mitochondrial myopathy was achieved in a more robust way in the study from Khan and colleagues. Deletor mouse model was treated with a precursor of vitamin B_3_ called nicotinamide riboside, which induced increase in creatine kinase, decreased accumulation of mtDNA deletions and increased OxPhos enzymes activities in skeletal muscle [41]. The suggested mechanisms of boosted mitochondrial biogenesis link to the activation of Sirt1 via stimulation of fatty acid oxidation (CD36, ACOX1 and MCAD) and increased UPRmt [41]. Following these findings, in a more recent study niacin, another vitamin B_3_ precursor, has been tested on patients with mitochondrial myopathy and mitochondrial biogenesis was observed together with boosted OxPhos function, and improved muscle strength and exercise performance [47]. This strongly supports the potential use of vitamin B_3_ precursors as exercise mimetics to facilitate the increase in muscle mitochondrial mass of patients with mitochondrial myopathy, since exercise interventions remain the most compelling method to activate PGC-1α signalling and mitochondrial biogenesis [125–127], but may be out of reach for patients with severe exercise intolerance and other complications.

Nevertheless, the risk to facilitate the clonal expansion of mutated mtDNA molecules by pharmacologically activating mitochondrial biogenesis in skeletal muscle remains. In patients with mitochondrial myopathy caused by mtDNA maintenance disorders or single, large-scale mtDNA deletion, it was observed that COX-deficient foci are characterised by locally increased TFAM level based on immunofluorescent labelling [16]. Moreover, Hsp60 and GPS2, which are proteins involved in the UPRmt signalling and known to promote both mitochondrial turnover and dynamics, were found at elevated level within COX-deficient foci, potentially driving the accumulation of multiple mtDNA deletion [128]. These findings strongly suggest that in the case of mitochondrial myopathy due to mtDNA mutations or deletions, the activation of mitochondrial biogenesis could be considered ‘safe' only if normal mtDNA molecules are replicated.

Alongside genesis of new mitochondria, old mitochondria are constantly being removed as a process that can happen as part of larger cellular degradation by autophagy or selectively by mitophagy. Autophagy has been documented in the skeletal muscle of patients carrying different types of mtDNA mutations [129–131] and nuclear variants [132], and has been linked to the myopathic features of these conditions. The presence of mtDNA deletions, more than point-mutation, seems to induce massive remodelling of the skeletal muscle tissue characterised by increased protein damage and ubiquitin-mediated proteasome activity, decreased amino acid salvage pathways and activation of autophagy [130]. Interestingly, there is evidence that autophagy is impaired in skeletal muscle from patients carrying the m.3243A > G variant, with down-regulation of pro-autophagy proteins (Beclin-1 and LC3-II) and up-regulation of autophagy inhibitor P-S6 [133]. The muscle-specific Cox15 KO mouse model has been shown to present defective autophagic flux in association to myopathic features in skeletal muscle [134]. Moreover, Mito and colleagues recently reported that in the Deletor mouse model, mitophagy actively contributes to the progression of mitochondrial myopathy following stage-wise dynamics. Normal fibres in Deletor mice are characterised by mitophagy that tends to localise around central nuclei and to a slightly lesser extent around peripheraly located nuclei. However, as the disease progresses, mitophagy begins to stall, with mitochondria accumulating in the subsarcolemmal region and ragged-ref fibres. When this happens, it can be noted that, whereas lysosomes had originally been preferentially located in the periphery of the muscle fibre, they later accumulate across its cytoplasm without a spatial pattern [135]. Similar findings were reported in tissues of patients with mitochondrial diseases [16,135]. Whether OxPhos-deficient fibres that have not yet accumulated abnormal mitochondria to become ragged-red fibres could have altered mitophagy, requires future studies.

Our knowledge on mitophagy's role on the onset and progression of mitochondrial myopathy is limited in human and most available animal models. However, extensive work has been performed around Pink1 and Parkin proteins in *Drosophila melanogaster*, mostly using specific reporters to assess mitophagy rate and function [136,137]. Parkin and Pink1 are involved in the regulation of both mitochondrial proteostasis and turnover, and decreased levels of these proteins have been shown to directly impair mitophagy in an age-dependent way [136]. Pink1 or Parkin knockdown (KD) in flies causes tissue-dependent disruption of mitochondrial proteostasis, increased oxidative stress, mitophagy suppression and mitochondrial aggregation in neuronal and muscle tissues, which can be rescued by overexpressing Nrf2 in the KD flies [138]. Moreover, mitophagy dynamics are age-wise dependent in Pink1 or Parkin KD flies, upon which mitophagy rate and number of mitolysososmes may change [138]. Conversely, overexpressing Parkin or Pink1 in the muscle of aged flies induced mitophagy and maintained proteostasis, thus rescuing OxPhos and ATP production, but it was observed to extend the lifespan of the files in an Atg1-independent manner, a key regulator of autophagy [139]. Together, these findings suggest a relevant interaction between autophagy and mitophagy in maintaining mitochondrial proteostasis in *Drosophila melanogaster* [139]. Interestingly, it was observed that the impaired autophagy and inhibited mitophagy observed in patients with mitochondrial myopathy can be ameliorated by rapamycin treatment, which is known to be a potent inhibitor of mTORC1 [39,134,135]. An increased mTORC1 signalling has been reported to orchestrate the metabolic remodelling seen in the skeletal muscle of these patients, therefore suggesting that the inhibition of mitophagy itself is orchestrated by mTORC1 signalling pathway, and that a rescue of the phenotype can be achieved by administering rapamycin [39–41,47]. However, available evidence in animal models of mitochondrial disease suggests that the modulation of mTORC1 and mitophagy are strongly dependent both on rapamycin dose administered and on underlying genetic cause [140]. Ongoing clinical trials on mitochondrial myopathy patients will elucidate this matter [141].

### Cellular processes

Mitochondria regulate several cellular processes by which signals are sensed and consequently processed to adjust and modulate metabolism to cellular needs. Here, we will discuss some of the most well-characterised processes, such as apoptosis, reactive oxygen species (ROS), Ca^2+^ signalling and ATP production, and will highlight some novel processes that may become important for the understanding of mitochondrial dysfunction in skeletal muscle.

Apoptosis is one of the first mitochondrial signalling pathway to be well-described, for which mitochondria are the executors due to their ability to release the pro-apoptotic protein, cytochrome c, into the cytosol through their permeability transition pore upon specific cellular conditions [142,143]. To be activated, this phylogenetically conserved mechanism requires the convergence of different mitochondrial signals, such as Ca^2+^ release and ROS production [144], and mitochondria morphology, including their network and the cristae morphology [145,146]. Skeletal muscle fibres are multi-nucleated post-mitotic cells that are resistant to initiate apoptosis to preserve their nuclear-to-cytoplasmic ratio [147]. However, it has been documented that under specific circumstances, such as atrophy or myopathy, a subset of myonuclei could undergo apoptosis [148]. In particular, a high rate of apoptosis was observed in skeletal muscle biopsies of single, large-scale mtDNA deletions and MELAS cases, where 34 000 fibres were analysed: apoptosis was observed only in ragged-red fibres, which were presenting with both mitochondrial proliferation and COX-deficiency [149]. Moreover, apoptosis was strongly associated with both high mtDNA mutation load and respiratory deficiency linking apoptosis with pathological markers of mitochondrial myopathy [149]. A significant correlation between apoptosis and high mtDNA mutation loads was reported in patients with encephalomyopathies too, where cytochrome c release and respiratory chain dysfunction can activate pro-apoptotic pathways and exacerbate the pathological mechanisms by inducing the removal of dysfunctional muscle fibres [150]. Studies from skeletal muscle pathology in patients with mitochondrial myopathy caused by other genetic aetiology found that apoptotic markers, including cytochrome c and Bcl-x, were localised in a granular distribution within the cytoplasm of either COX-negative fibres or ragged-red fibres, and DNA breaks, which are signs of activated apoptosis, were observed both in the myonuclear and mtDNA genome [151,152]. However, another study evaluating skeletal muscle biopsies of patients with mitochondrial myopathy, highlighted the extreme variability of apoptotic markers in myopathic muscle [153]. This might be related either to the heterogeneous nature of mitochondrial diseases [154], or to the fact that apoptosis may not always be executed due to a downstream blockage [155]. Indeed, patients with mtDNA mutations or depletion syndromes, caused by autosomal [156,157] or X-linked [158] mutations have been reported to have a propensity for skeletal muscle fibre apoptosis, suggesting that the interlink between mitochondrial dysfunction and apoptosis may also depend on nuclear genome variants or genetic background more generally.

ROS consists of radical and non-radical oxygen species, usually formed by partial reduction in oxygen during OxPhos [159]. Superoxide anions (O^2−^) are generated by electron leakage at CI, into the mitochondrial matrix, and at CIII into both the mitochondrial matrix and intermembrane space [160,161]. Under physiological conditions, mitohormesis maintains ROS production, and therefore oxidative stress, within certain levels that induce beneficial health outcomes, such as metabolic health and longevity [162]. However, impaired OxPhos function in skeletal muscle will increase oxidative stress and induce a cascade of downstream events, such as modulation of transcription factors mitochondrial biogenesis, and myogenesis [163,164]. For this reason already back in 1997, Rosenberg and colleagues proposed that reduced mitochondrial ATP production and increased mitochondrial production of ROS were both two sides of the same phenomenon, such as oxidative phosphorylation defect that leads to the onset of symptoms in mitochondrial disease patients in general [165]. To test the hypothesis, an Ant1 KO mouse model was generated, where the adenine nucleotide transporter isoform 1 was lacking in the IMM of mitochondria in both heart and skeletal muscle; this would induce not just the blockage of ADP/ATP exchange between mitochondria and cytosol, but also consequent OxPhos defects [166]. In Ant1 KO mouse model, skeletal muscle indeed presents with myopathic features, decreased OxPhos function and an associated increase in ROS production through increased expression of MnSOD and Gpx1 proteins [166]. Similarly, skeletal muscle fibres of patients with mitochondrial encephalomyopathy present overproduction of ROS with a significant increase in MnSOD [167,168], which significantly correlates with COX-deficiency [152,169]. Moreover, the mtDNA mutation rate is increased in Ant1 KO mouse model compared with controls, probably because mutations arise earlier during the life of these mice, highlighting the fact that the amplified oxidative stress is likely to contribute to the observed accumulation of mutated mtDNA molecules [166]. So far, the study of these intertwined mechanisms has been challenging, partly because mammals do not share the same mechanism of ROS production observed in simpler organisms such as *S. Cerevisiae* [170,171]. What is known is that ROS overproduction acts as a disease modifier for the progression of mitochondrial myopathy by affecting several different pathways involved in mitochondrial turnover and other cellular processes, such as satellite cells differentiation and tissue regeneration [172–174]. For instance, in a model of induced pluripotent stem cells obtained from fibroblasts of patients with mitochondrial encephalomyopathy, ROS overproduction was demonstrated to promote both autophagy and mitophagy, inducing a decrease in OxPhos function and cell viability [175].

Mitochondria are central for Ca^2+^ homeostasis. Ca^2+^ is a ubiquitous second messenger, usually kept as a free ion at very low cellular concentrations and mainly compartmentalised in either the endoplasmic reticulum or mitochondria [176]. This is mainly due to the fact that Ca^2+^ concentration ubiquitously regulates the turnover of metabolites involved in the TCA cycle (e.g. pyruvate and α-ketoglutarate), and modulates physiological enzymatic activities, such as glycerophosphate dehydrogenase, malate-aspartate shuttle enzymes, aspartate-glutamate carriers [177], and matrix dehydrogenases [178]. Importantly, the concentration of Ca^2+^ regulates ATP synthesis [179,180] and can induce apoptosis [181]. Although Ca^2+^ stored in mitochondria can be unselectively released into the cytoplasm during apoptosis and via the permeability transition pore [181], we now know that Ca^2+^ uptake and concentration in the mitochondrial compartments are regulated by different channels and transporters in the outer and inner mitochondrial membranes [182]. We also need to remember that Ca^2+^ is essential for excitation-contraction coupling during skeletal muscle contraction, for which mitochondria are the major source of both ATP and Ca^2+^ buffering, through sustaining OxPhos function and mitochondrial fusion, respectively [89].

Ca^2+^ enters the mitochondria through voltage-dependent anion channels (VDAC) are porin-like proteins of the mitochondrial outer membrane that facilitate the entry of metabolites and ions, including Ca^2+^, into the mitochondrial intermembrane space [183]. Skeletal muscle from patients with mitochondrial myopathy usually presents with a substantial decrease in VDAC, which implies a potential impairment of their Ca^2+^ uptake capacity[21,31–33]. However, the channelling towards the mitochondrial matrix is performed by the mitochondrial calcium uniporter (MCU), which involves different components, such as MICU1 [184]. Although loss of function of the MCU spares mitochondrial respiration and membrane potential, it decreases the mitochondrial Ca^2+^ uptake and attenuates the activation of the TCA cycle [184]. Similarly, loss of MICU1 impairs MCU function inducing decreased ATP production, increased autophagy, and elongated mitochondria within the sarcomere of cardyomyocites [185]. In recent years, it has been demonstrated that loss of MICU1 increases the resting Ca^2+^ concentration in mitochondria with activation of the TCA cycle enzyme PDH and Drp1 protein, resulting in increased mitochondrial fission [186]. Specifically in skeletal muscle from myopathic patients, the role of MICU1 has been validated as the primary determining factor in maintaining Ca^2+^ basal concentration and preserving both mitochondrial morphology and metabolism [187].

Beside the mechanisms described above, most of Ca^2+^ that enters mitochondria derives from their contact sites with the endoplasmic reticulum, known as mitochondria-associated membranes [188,189]. In skeletal muscle, the sarcoplasmic reticulum creates junctions with mitochondria, which bring together the sarcoplasmic reticulum, and the mitochondrial outer and inner membranes, to provide a favourable spatial alignment between these structures [190]. To date, it is not yet known what happens in skeletal muscle of patients with mitochondrial myopathy. However, a link between CV deficiency and sarcoplasmic reticulum distress has been recently described in skeletal muscle from patients with tubular aggregates myopathy, suggesting that OxPhos defects might have an association with the impairment of mitochondria-associated membranes formation [191]. The associated affected Ca^2+^ microdomains within or in proximity of COX-deficient fibres might function differently to induce different contraction patterns compared with normal fibres, triggering downstream dysfunctions [192]. In a muscle-specific Drp1 KO mouse model, it was reported that the deletion of Drp1 could induce changes both in mitochondrial dynamics and turnover, and in Ca^2+^ microdomains and homeostasis, inducing aberrations in OxPhos functions, and UPR both in mitochondria and in the myofibres, with downstream global reduction in skeletal muscle mass [193].

The sodium/lithium/calcium exchanger (NCLX) actively maintains the Ca^2+^ concentration of the mitochondrial matrix within physiological levels by pumping Ca^2+^ into the mitochondrial intermembrane space [194]. Indeed, the function of the NCLX goes beyond mere import/export of ions as it also interacts with other inner membrane proteins. In cardiomyocytes it was observed that NCLX and sarcoplasmic/endoplasmic reticulum Ca^2+^-ATPase (SERCA) co-localise at the contact points between mitochondria and the sarcoplasmic reticulum, creating a spatial advantageous cross-talk for Ca^2+^ exchange [195]. Since all SERCA isoforms are co-localised with NCLX, it is thought that a similar co-localisation happens in skeletal muscle too [187]. Finally, it is worth mentioning that, although a central role for mitochondria in Ca^2+^ homeostasis has long been recognised in other neuromuscular disorders [196], not much is known for mitochondrial myopathies specifically. Cells from patients with mtDNA mutations affecting tRNA^Lys^ were found to have OxPhos deficiency due to pathogenically driven impairment in mitochondrial Ca^2+^ homeostasis; this phenotype could be restored by administration of drugs targeting the mitochondrial Ca^2+^ signalling with subsequent increased ATP production [197]. Moreover, mutations of CII subunits in fibroblasts from patients with Leigh syndrome have been linked to mitochondrial dysfunction and differential metabolic arrangements depending on Ca^2+^ signalling downstream mechanisms [198]. Further investigations will help to understand in detail the role of mitochondrial Ca^2+^ homeostasis in mitochondrial myopathy pathogenesis.

Mitochondria are super dynamic organelles able to release their circular mtDNA molecules to transmit signals within the cell and between different types of cells, therefore extending their functions beyond the mitochondrial double membrane. It has been known for decades now that the mtDNA copy number detected in a cell is in excess compared with the amount required to sustain mtDNA transcriptions and translation of mtDNA-encoded OxPhos proteins [199]. A novel suggestion is that the excess mtDNA copies function as sensors for both cellular and mitochondrial homeostasis [200]. The mtDNA molecules are usually packaged into nucleoprotein complexes where TFAM is the most abundant protein and binds mtDNA in a non-specific way [201]. Usually, mtDNA is localised in the mitochondrial matrix; however, under certain circumstances it can be released into the cytosol. To date, we know that the extrusion of mtDNA from the mitochondrial matrix could be either performed by the oligomerization of VDAC, under oxidative stress conditions [202], or through the permeability transition pore, during apoptosis [203]. However, in skeletal muscle of patients with mitochondrial myopathy, mtDNA can be released via a non-specific mechanism due to swelling of mitochondria and rupture of the double membranes [97].

One of the best-characterised processes activated by the release of mtDNA is the activation of the inflammasome, which is a multi-protein complex able to induce the secretion of inflammatory cytokines [204]. It is important to highlight that the nature of mtDNA released in the cytosol might differ and involve already existing mtDNA molecules, newly synthetised [205] or consist in oxidised mtDNA released fragments [206]. Mutations of mtDNA-encoded cytochrome b in patients presenting with fibromyalgia have been associated with patient symptoms and activation of the inflammasome [207], highlighting the possibility that this could also be the case in skeletal muscle of patients with mitochondrial myopathies. However, mtDNA can be detected in a circulating cell-free state in different bio-fluids of healthy individuals [208]. Several studies seem to reveal that the biological reason behind its presence is not immunological as it is for mtDNA released into the cytosol [208]. These findings suggest that circulating cell-free mtDNA signalling may have a paracrine and endocrine role. An example can be given by the signalling between damaged skeletal muscle fibres and satellite cells during the process of muscle regeneration. An emerging role in skeletal muscle maintenance has been attributed to mitochondrial-derived vesicles (MDVs), which complement the mitochondrial quality control processes by cooperating with mitophagy to preserve mitochondrial homeostasis [209,210]. Currently, there is no evidence of the role of MDVs in the pathogenesis of mitochondrial myopathy in skeletal muscle. However, MDVs establish the link between damaged skeletal muscle fibres and regenerative satellite cells by transporting a cargo of mtDNA molecules, proteins, and metabolites [211,212]. Conversely, it has been observed that muscle stem cells can deliver MDVs to skeletal muscle fibres affected by mitochondrial dysfunction: the cargo proteins are specifically delivered to the damaged skeletal muscle fibres within 2 hours of mitochondrial dysfunction induced by hydrogen peroxide treatment. Moreover, these cargo proteins have been found to co-localise within the dysfunctional mitochondria to eventually revert the phenotype observed [213]. Further investigations could clarify the MDVs role in mitochondrial myopathies and their potential for novel treatment avenue.

### Muscle cell morphology and function

Skeletal muscle fibres are made up of myofibrils composed of repeating sarcomeres along the length of the myofibrils. Mitochondria in turn are sandwiched between the myofibrils at the *z*-band of each sarcomere and around the periphery of the cell below the sarcoplasmic membrane. In *Drosophila melanogaster*, mitochondrial morphology has been demonstrated to impact on myofibrillar morphology and specifically myofibrillar branching [214]. Researchers found that the transcription factor Spalt communicates to the mitochondria in muscle fibres that they should intercalate between the myofibrils, and that in turn, the myofibrils will provide a mechanical constraint on their morphology [214]. The muscle selector Spalt controls a morphological switch between different muscle types in *Drosophila melanogaster*, and inducing changes to its signalling has been shown to cause a conversion between different muscle types [215]. Work comparing the morphologies of myosin fibrils and mitochondria in different organisms (drosophila, mice and humans) has found that the cross-sectional area of myosin fibrils varies along their length and is smaller at the z-band where mitochondria are positioned [216]. Furthermore, the myosin fibrils curve with their highest curvature associated with mitochondrial contact suggesting that myosin filaments and myofibre structure are impacted by both mitochondria position and morphology [216]. In ragged-red fibres of patients with mitochondrial myopathy, the increased subsarcolemmal and intermyofibrillar mitochondrial masses are clearly associated with sarcolemma distension and disruption of myofibrillar organisation, respectively [97].

Myofibrillar branching is thought to be important for the ability of a muscle to generate force and reduced branching is believed to reduce contractile force. This relationship has also been demonstrated in healthy human muscle and, together with work in *Drosophila melanogaster*, suggests that inducing mitochondrial fusion leads to a reduction in myofibrillar intercalation. This would suggest that low levels of mitochondrial dysfunction are likely to disrupt myofibril branching and therefore skeletal muscle function [217]. It is also possible that increases in mitochondrial mass as we see in ragged-red fibres would have a similar impact. Work is needed to fully characterise the relationship between mitochondrial function and myofibrillar morphology and function.

It would remiss here not to address the long debate about the role of mitochondrial dysfunction in both muscle atrophy and sarcopenia. Whilst mitochondrial dysfunction has been demonstrated to be intricately linked with mitochondrial morphology, as described above, mitochondrial morphology has further been shown to impact muscle mass [218]. In sarcopenia, dysfunction of mitochondrial fusion leads to muscle fibre atrophy and decline in muscle mass, as it is associated with reduced mitochondrial fission and fusion machinery [30,219,220]. Furthermore, mitochondrial fission is important for muscle development, maintenance, and function. Mitochondrial morphology has also been shown to have further knock-on effects in muscle including impacts on muscle inflammation [221]. Therefore, the impact of mitochondrial dysfunction on mitochondrial morphology may further trigger these downstream processes. Moreover, while data in rats suggest that high levels of mtDNA deletions and mitochondrial dysfunction are causative of muscle fibre atrophy, only a small proportion of fibres with mitochondrial dysfunction are atrophied [222]. Indeed, given the much higher levels of mitochondrial dysfunction in patients with mitochondrial diseases, when compared with older healthy individuals, if mitochondrial dysfunction was driving muscle atrophy, we would expect to see much more atrophy in patients, which is not the case [223,224].

Finally, central nuclei are a well-known early pathological hallmark in mitochondrial myopathy shared with many other myopathic diseases. These have been suggested to be the nuclei from satellite cells that fuse with muscle fibres, and which migrate to the centre of the fibre before being redistributed to its periphery, meaning that increased central nuclei could simply be a marker of increased fusion of satellite cells during myopathic muscle repair. However, Mito and colleagues found clusters containing lysosomes, mitochondria, and mitolysosomes adjacent to the central nuclei of skeletal muscle fibres from Deletor mice [135], which looked remarkably similar to findings of nucleophagy in genetic nuclear encephalopathies causing myopathy [225]. Therefore, whether centralised nuclei are linked to mitochondrial dysfunction in mitochondrial diseases with disease-specific characteristics warrants further investigation. One possibility is that the starvation-like response triggered by mitochondrial myopathy, which induces the remodelling of the one-carbon metabolism and ISRmt disturbing cellular dNTP pools [36,39,40], may trigger nucleophagy in an attempt to regain homeostasis. Further to this, observations in immunofluorescent labelled skeletal muscle sections from patients with mitochondrial myopathy suggest a disorganisation of myonuclei, for which it is not yet clear whether the underlying mitochondrial dysfunction has a role.

## Conclusions and future perspectives

Mitochondrial and skeletal muscle biology are profoundly intertwined, and understanding their physiological mechanisms will allow the underpinning of pathological processes underlying the onset and progression of muscle disease throughout the lifespan of patients affected by mitochondrial disorders. Over the last decade, giant steps have been made towards the dissection of metabolic remodelling in mitochondrial myopathies, although highlighting the fact that the adaptations observed are inevitably restricted to the subgroup of mitochondrial myopathy investigated. The advent of more sophisticated technologies and methodologies of analysis made it possible to look closer to the structure and morphology of mitochondria within skeletal muscle fibres, showing how their localisation within cells and tissues has a powerful and bidirectional influence in the functional output of the organelle, the cell and, ultimately the muscle, in both health and disease. Research into mitochondrial myopathies is consequently challenging, not just due to their heterogeneous genetic causes and inheritance patterns (mtDNA/nuclear variant, mtDNA point mutation/mtDNA deletion), as well as complex dynamics (homoplasmy/heteroplasmy, mtDNA copy number); but also because mitochondrial populations have compartmentalised biological skills based on their cellular localisation (subsarcolemmal, intermyofibrillar, perinuclear) and in close relationship with skeletal muscle fibre-type composition, biology and function (e.g. extra-ocular muscles, proximal limb muscles, or the diaphragm). In our perspective, filling these knowledge gaps, which are highlighted in more detail throughout this review, requires targeted research to advance our understanding of how pathological changes in mitochondria and muscle lead to clinical symptoms and progressive disease. To date, many animal models, mostly mouse models, have been generated to mechanistically study mitochondrial myopathy. However, and despite their unquestionable research value of these models, acknowledging their limitations due to species-related biological differences will be essential to combine findings from animal, human and cell culture models.

## Open Access

Open access for this article was enabled by the participation of Newcastle University in an all-inclusive *Read & Publish* agreement with Portland Press and the Biochemical Society under a transformative agreement with JISC.

## Abbreviations

AICAR: 5-aminoimidazole-4-carboxamide ribonucleoside
Ca^2+^: calcium anion; CI-V, complexes I-V
COX: cytochrome *c* oxidase
IMM: inner mitochondrial membrane
ISRmt: integrated mitochondrial stress response
KD: knockdown
KI: knock-in
KO: knockout
MCU: mitochondrial calcium uniporter
MDVs: mitochondrial-derived vesicles
NCLX: sodium/lithium/calcium exchanger
O^2^: superoxide anion
OMM: outer mitochondrial membrane
RIRCD: reversible infantile respiratory chain deficiency
ROS: reactive oxygen species
SERCA: sarcoplasmic/endoplasmic reticulum Ca^2+^-ATPase
TCA: tricarboxylic acid
UPRmt: mitochondrial unfolded proteins response
VDAC: voltage-dependent anion channels
